# Fear, faith and finances: health literacy experiences of English and Swahili speaking women newly diagnosed with breast and cervical cancer

**DOI:** 10.3332/ecancer.2022.1350

**Published:** 2022-01-27

**Authors:** Dinah Kassaman, Tayreez Mushani, Peterson Kiraithe, Sharon Brownie, Margaret Barton-Burke

**Affiliations:** 1 School of Health and Society, University of Salford, Mary Seacole Building, Frederick Road, Salford M6 6PU, UK; 2 School of Nursing and Midwifery, Aga Khan University, Nairobi, Kenya; 3 School of Nursing, Midwifery & Public Health, University of Canberra 11 Kirinari St, Bruce, ACT 2617, Australia; 4 School of Medicine and Dentistry, Faculty of Health, Griffith University, 1 Parklands Drive, Southport 4215, Australia; 5 Centre for Health & Social Practice, Gate 3 Tristam Street, Waikato Institute of Technology, Hamilton 3200, New Zealand; 6 Memorial Sloan Kettering Cancer Center, 1275 York Ave, New York, NY 10065, USA; 7 Rutgers Camden School of Nursing, 530 Federal St, Camden, NJ 08102, USA; a https://orcid.org/0000-0001-6078-4626; b https://orcid.org/0000-0001-7204-2451; c https://orcid.org/0000-0001-7816-5911

**Keywords:** health literacy, breast cancer, cervical cancer, newly diagnosed cancer, health information

## Abstract

Breast and cervical cancer are among the leading causes of cancer-related deaths globally. In Kenya, delayed presentation and diagnosis contribute to breast and cervical cancer mortality. The Kenyan government acknowledges the cancer burden with estimated 39,000 new cases diagnosed and 27,000 deaths per annum. Mortality can be reduced if cancer is diagnosed early and with appropriate treatment. Health Literacy (HL) about cancer screening, diagnosis and treatment is important in reducing mortality, but there is little understanding about HL levels, experiences of patients diagnosed with breast and cervical cancer and the contexts in which they make decisions. In this study, health literacy is defined as the degree to which individuals have the capacity to obtain, communicate, process and understand basic health information and services needed to make appropriate health decisions. This exploratory qualitative study investigated the HL experiences of accessing and using health information in women with any stage of breast or cervical cancer presenting at the Aga Khan University Hospital (private) or Kenyatta National Hospital (public) in Nairobi, Kenya. Data were gathered through semi-structured interviews from a purposive sample of 18 women. Interviews were transcribed verbatim, and the Consolidated criteria for reporting qualitative studies guidelines guided data analysis. The findings may aid development of patient education tools and determine effective ways of communicating cancer-related health information to improve the knowledge and health-seeking behaviours of Kenyan women. This project identified sociocultural beliefs and factors that influence how women understand information provided by healthcare professionals. Themes that arose included but were not limited to: *fear, despair and agony at diagnosis, faith, social support, side effects, cancer-related stigma and financial burden of cancer as a barrier to getting information*.

## Background

The World Health Organization reports that global mortality from cancer exceeds that of HIV, Malaria and Tuberculosis combined [1]. Furthermore, the rise in non-communicable diseases including cancer is burdening Lower-Middle Income Countries such as Kenya, at a greater rate than the western world [1]. Government leaders and Ministry of Health officials in Kenya acknowledge the increasing burden of breast and cervical cancer and the need to strategically focus resources on prevention, early detection and treatment [2, 3]. The government identifies cancer as a priority and is committing resources to prevention, detection and treatment; however, without research about health literacy (HL) in Kenya, the limited resources may be ineffective to the nation.

Cancer is the third leading cause of death in Kenya with 70%–80% of cancers diagnosed in late stages of the disease [4, 5]. The Kenyan government acknowledges the cancer burden with estimated 39,000 new cases diagnosed and 27,000 deaths per annum. Breast and cervical cancer have rates of 23.3% and 20%, respectively, and this incidence may be attributed, in part, to a lack of HL and cancer awareness [4, 5]. The Institute of Medicine defines health literacy as ‘the degree to which individuals have the capacity to obtain, communicate, process and understand basic health information and services needed to make appropriate health decisions’. Thus, HL is a public health issue [5, 6], with inadequate knowledge identified as a barrier to cancer screening in Kenya [6]. With poor knowledge about screening, risk factors, symptoms, signs, treatment and prevention of cancers reported [6, 7], there is a need for increased patient awareness and educational programmes to improve effective diagnosis and management of cancers [7]. While studies related to cancer patient education [8, 9] highlight the need for increased patient education and effective tailored teaching programmes [10], there is a paucity of research on cancer-related HL to inform current health care in Kenya. Significant changes in global healthcare delivery over the past few decades have caused a shift towards a patient-centred care [11]. This means that patients need an understanding of health information to make important decisions about disease prevention, screening and treatment. However, patient-centred care can be compromised when health care providers cannot draw upon contextualised research [2] to understand HL. This paper is part of a wider study which explored, assessed and consolidated cancer-related HL information across the illness trajectory among women diagnosed with cancer of the breast or cervical in Kenya. In this study, we focused on how some of the sociocultural beliefs of Kenyan women affected their understanding of the information provided to them by healthcare professionals throughout the cancer journey.

Hence, this study aimed at examining the role played by (some) socio-cultural beliefs and support in the women’s understanding of the information provided to them by healthcare professionals throughout their cancer journey.

## Methodology

A purposive sample of 18 women receiving treatment at the Aga Khan University Hospital or Kenyatta National Hospital and treatment centre in Nairobi was sought for a three-stage interview process, specifically: at diagnosis, a follow-up interview at 4–6 weeks post diagnosis and a final interview at 12 weeks post diagnosis. Women were invited to participate in the semi-structured interviews if they were of Kenyan descent, diagnosed with stage 1–4 of breast or cervical cancer as a primary malignancy and spoke English or Kiswahili (Kenyan National language). An interview schedule (Appendix A) guided the data collection. The second and third interviews were informed by the findings from the first interview and were tailored to the individual participants. The original semi-structured questions and prompts explored the personal experience of a woman undergoing cancer treatment, her cancer information needs, the impact on her family and/or community and her thoughts about health information. Interviews were conducted in English and Swahili, audiotaped, transcribed and reviewed by the investigators for accuracy. Ongoing analysis continued between each in-depth interview. Preliminary notes, themes, hunches, interpretations and ideas were written in a notebook and became part of the data. All tapes were transcribed, reviewed and amended to include verbal and non-verbal cues such as pauses, laughter or other emotion that were expressed during the interviews. Conduct of the study and reporting of results followed standards for qualitative research (Standards for Reporting Qualitative Research).

Ethical approval was obtained from the University of Salford Ethics committee, the Institutional Ethics Review Committees of the Kenyatta National Hospital and Aga Khan Hospitals, and a research permit from the National Commission for Science, Technology and Innovation (NACOSTI). The Participant Information Sheet (PIS) and informed consent (Appendix B) explained the purpose of the research, the benefits and any anticipated risks. The consent to use their information to publish was also included. Confidentiality, anonymity, privacy, dignity, respect and trust were observed. Anonymity was achieved by having no names or any form of identification written on interview guide; only number codes were used to protect the subjects. Partici pants had the right to withdraw any time without giving any explanation or not answering a question one was not comfortable with. An interview guide (Appendix B) was designed to explore and describe HL regarding breast and cervical cancer within a Kenyan context. Structured questions focused on a woman’s sources of information, understanding and beliefs about cancer, interpretation of the information provided by health care professionals concerning diagnosis and planned treatment and perceived educational needs.

### Data analysis

The data analysis, write-up and publication of this research project followed the recommendations in the Consolidated criteria for reporting qualitative studies Guidelines [12]. Data analysis had three components – initial, ongoing and final. Initial data analysis began while listening in the interview for conversational elements like inconsistent, vague or cryptic comments. Analysis criteria for the interviews included frequency, extensiveness, intensity and specificity. Interviewers sought a depth of responses by having the woman describe the personal context for her response. Study team members read transcripts and listened for 1) Frequency – how often or frequently is a point being made; 2) Extensiveness – how extensively does she describe specific aspects of provided health information; 3) Intensity – how intensely are certain topics discussed and 4) specificity – how specific is the discussion about the health information. Final analysis includes analysing data (tapes, transcripts, notes) as complete data. The data were reviewed for within interview findings, across-interview findings and between-interview findings. The analysis ensured the reliability, validity and trustworthiness of the data and the study conclusions.

## Findings

### Participant demographics

The sample consisted of 18 Kenyan women diagnosed with breast and cervical cancer being treated at either Aga Khan or Kenyatta National Hospital in Nairobi, Kenya.

### Study sample description

Table 1 shows the sociodemographic characteristics of the participants. From the original sample, two women died after the first interview and one withdrew. Their interview data were not included in the final analysis.

### Main findings

There were six themes that emerged from the study data. The terms *faith, fear* and *finances* were themes that were mentioned by most of the women in the sample. Other themes were items *like stigma, social support* and *side effects of treatment*. The key quotations included are brief but are the powerful words the women used as they shared their perspectives. The participants are identified based on the hospital they attended (public or private) and their diagnosis (breast or cervical cancer) (Table 2).

#### Faith

In this study, faith was consistently mentioned by all participants. Faith is defined as a belief in a higher spiritual being [13]. The term faith is frequently interchanged with the term religion, which refers to a belief system. Faith can likewise mean trust in a non-religious entity and includes the commitment an individual has towards oneself, other individuals and the universe to practice what they believe in [14]. Spirituality goes hand in hand with faith and it refers to the reason of one’s existence, the meaning and values to which one ascribes [13].

The women understood faith to be important but also saw it as central to their cancer diagnosis and treatment. They consistently believed that God was their guide. They believed that God gives the disease and would provide a cure/treatment. This belief was the reason for their delay after discovering the lump.

*My daughter told me, No mum. There is God in heaven, it shall be well. So, even that is what made me to stay for all that long not taking any action towards that lump (Private hospital participant 1 with breast cancer)*

Cancer was seen as an opportunity to trust and demonstrate how God enables patients to find meaning in their suffering. Participants lived with hope for the future and trusted in the mighty power of God even when everything seemed futile. A majority of the participants alluded to their faith as a major coping strategy once they were diagnosed with cancer. Thus, suggesting that faith was also a support mechanism; women consistently referred to God as a guide, protector and helper throughout the treatment journey.

*God is there, I remember there is somebody, there is somebody who told me that God is there (Private hospital participant 6 with breast cancer)*

Participants mentioned the threat to their well-being that comes with a cancer diagnosis along with the challenges related to treatment, but the faith that God would not leave them at their time of need was comforting. The sense of trust, hope and reliance in their faith gave these women strength to cope immediately after a cancer diagnosis.

*…..But because I am a Christian, I said even though I am going to die ... I am not going to be afraid (Private hospital participant 1 with breast cancer).*

Individuals who encounter difficult situations like a cancer diagnosis seem to benefit when using their faith and belief in God. One woman recalled the time the doctors at a mission hospital broke the news that she had cancer. Their reference to God resonated well with her faith.

*… we know you are a Christian and you really pray God and I said ooh yes, I believe in my Lord Jesus in all my life, and I thank him even for whatever am going through right now……… so the doctor said that is very good and even its encouraging and that is now when they started analyzing the results. They told me Mama the biopsy results have cancer but mama do not worry, thank God, we are going to treat you. I said yes (Private hospital participant 5 with breast cancer).*

The women in the study were from both Muslim and Christian backgrounds and they described a unique connectedness to God after the cancer diagnosis. Participants had a positive attitude stating that their faith had grown after the cancer diagnosis. They quoted verses that gave them hope and encouragement.

*And also he then tells me, I should not be troubled I my heart but is should trust God, so I remembered …and also in Psalms 12:6 he says even when I pass in the valley of the shadow of death, the shadow of death I shall not fear, because the Lord is there, he comforts me. All along, I have experienced strength, which is not mine. So I have learnt to stand still and see the salvation of the Lord (Private hospital participant 5 with breast cancer).*

*But I told God you are the one I put my trust in you for I believe that you will heal me.*

*I only say one word that doctor treat but God heals. (Public hospital participant 7 with breast cancer)*

A participant from the Muslim faith stated:

*I just say it was God that I just landed to right hand so that the journey actually is easy. …..but I thank God because am not in pain (Private hospital participant 7 with breast cancer).*

#### Fear

Fear permeates the transcripts experienced by most women in this study. In this study, fear is defined as a feeling of apprehension about an event or the outcome of an event [15]. Fear is seen in four different forms, fear at finding a lump, fear of death, fear of recurrence and fear of unknown. It should be noted that participants knew that cancer was a fatal disease that would lead to death and in their fear some used Christian scripture, stating that believers do not die.

Participants exhibited fear when they first identified a lump in their breast for they associated with breast cancer.

*……………… I could tell they have found some cancer in the breast, so that’s why I was anxious I wanted the doctor now to tell me whether the lump was cancerous or not (Private hospital participant 3 with breast cancer).*

*…………the first day I talked with my daughter because I just received a call informing me that a lady hanged herself because she has found she has a lump in her breast. So that moment I just stood up in front of my daughter and removed my blouse so, since in knew how to check on my breast I started doing checking. So it reached to a point I realized I have a lump and I told my daughter……… I called her by her name W! Imagine I have got a lump in my breast. Should I take a rope and hung myself (Private hospital participant 1 with breast cancer).*

Breast cancer magnifies ones’ fear due to the location and symbolic nature of the breast [16]. The breast and cervical are termed private parts of the body and organs of posterity in the Kenyan culture. When women are faced with illness to these private parts, they are more likely to experience fear and anxiety.

*……When I was told I was positive, I was shocked and even cried. Because I thought I was dead. You know when we were schooling we were just saying, ‘cancer is a killer disease’. At that hour I saw, I was dead!..... (Private hospital participant 2 with breast cancer).*

*……….when I was told I have cancer. In the first day it was very scary, very scary, and inside me I tried to think about it, I have heard about cancer. I have heard people dying of cancer and now I have the breast cancer …… (Private hospital participant 5 with breast cancer).*

Study participants expressed fear of recurrence of cancer during their interviews especially as they underwent their treatment. Some participants had challenges and delays in accessing treatment on time also expressed the fear of the metastasis of the cancer.

*…….I was worried as much, as this is a disease, because of such disease people fear cancer and I was afraid it’s multiplying…………….I was very shocked*

*..(Private hospital participant 9 with cervical cancer).*

*……….Am saying damages like you see, like this lump, I have been late for drugs it seems like I will be amputated the entire organ. And also sometimes I find myself swollen at my armpits and those are the damages I have witnessed about it………. (Public hospital participant 2 with breast cancer).*

Fear of the unknown is a type of fear that occurs in an individual when faced with uncertainty [17]. The fear of unknown was evident across study transcripts and was expressed as a result of women not knowing what caused the cancer, how it can be prevented from recurring and how treatment will affect them. Many women with advanced disease are more fearful of the treatment than the disease itself when they are not aware of the treatment procedures and the outcome.

*………When I shared with my husband everybody was that there was a lot of fear, colleagues a lot of fear. It’s only that one would not tell you outright that that was cancer bout there was a lot of fear from whatever expression you would tell exactly it was cancer…and the treatment is complicated……. (Private hospital participant 3 with breast cancer).*

#### Finances

Cancer treatment is expensive, but treatment is especially difficult when patients are diagnosed in sub-Saharan Africa where majority live on less than a dollar per day [18]. Finances and the financial burden of cancer could be found in almost every interview. Finances, in this study, are defined as the amount of money needed to pay for diagnosis and treatment, as well as the costs necessary to pay for incidental items such as transportation to the hospital, food during treatment and items that insurance does not cover. Participants stated:

*……..I was shocked because I didn’t expect to have cancer. I got shocked for I knew my situation is bad for being the breadwinner now my life is ended with cancer….. (Public hospital participant 4 with breast cancer).*

*One was talking about the money that, it is expensive, cancer is an expensive disease (Private hospital participant 10 with cervical cancer).*

Participants talked about health insurance during diagnosis and treatment and a failure to adhere to their treatment schedule as they were short of finances to cover the treatment costs. Patients made a habit of calling each other if they did not come for treatment.

*There was one patient that we started chemo with, I normally take the phone numbers of other patients and do call them. So I called her the first time when I saw she had not come the first time, the second time I never saw her so I called her and asked her what was the problem and she told me, my friend, it’s not my will, I could have desired to come but money is an issue for buying those drugs. Even the bus fare to come to the hospital I do not have (Public hospital participant 1 with breast cancer).*

It is important to understand the health insurance within Kenya and how that insurance finances the costs for women diagnosed with breast or cervical cancer. All study participants had the National Hospital Insurance Fund (NHIF) coverage that took care of some treatment costs. There were participants with NHIF but there were others whose NHIF was not current and had problems in meeting their treatment cost. Overall, cancer patients in Kenya have various levels of insurance coverage ranging from no coverage to comprehensive coverage [19], all of which impact treatment outcomes.

*…. When I ask others for the help, others tell me to use the NHIF card ………… {participant talked very softly that the statement couldn’t be audible) (Public hospital participant 4 with breast cancer).*

*Then the other lady sitted next started to explain to me the issue of Texas telling me to go Texas when I come and they say that there are no drugs in Kenyatta I just go to Texas and I will fill the NHIF form and I will be issued with the drugs. Then I saw that the discussion helped to know that. (Public hospital participant 4 with breast cancer).*

Some participants reported having missed their treatment for they had not/could not pay for NHIF insurance premiums, which jeopardised their treatment schedules. They also reported a recurrence of the cancer after finishing their scheduled treatment plan or due to interference of their treatment schedule by lack of funds to meet treatment cost

*‘It is not really what the disease per se but the expense, it is a very expensive disease to treat…….. the lady kind of what stopped treatment because she what kind of said what she couldn*’*t afford what a treatment (Private hospital Participant number 7 with breast cancer).*

One participant delayed to start treatment because of a doctor’s strike at the public hospital.

*I had breast pains and something had swelled inside. At that time, there was a doctor strike and I was much troubled there. I was a bit in pain but was prescribe some pain killers by the doctor. After the biopsy, the results came out and I picked results, when results were to be read to me it was a problem for there was no doctor to read for me for about three weeks without results being interpreted to for me. After that, the doctors resumed from the strike and I come and results were interpreted to for me. When I come I did not have money, they wanted one thousand one hundred shillings (USD 11) which I did not have at that moment. (Public hospital participant 4 with breast cancer).*

*Because my husband told me that we had also to go for the hospital. To go to the hospital. But we didn*’*t had money. So we were waiting for the time maybe when we would have money. And then I would go to the hospital (Private hospital participant 10 with cervical cancer).*

The costs of cancer were direct and indirect. Some of the direct costs for cancer treatment included women purchasing needles for diagnostic biopsy and the cancer chemotherapy. Once again the costs outspent the costs of living in Nairobi, Kenya [19]. The indirect costs of cancer included transportation to and from the hospital and the hospital stay.

*They told me that the treatment of cancer is a bit high it cost a lot of money. The doctor told me now you just go and break the news to the people that would help you, maybe your family because the cost of treatment of cancer is a bit high. It will cost you much. I didn*’*t know what to do (Private hospital participant 10 with cervical cancer).*

A cancer diagnosis and treatment were highlighted as a financial strain with financial support often needed as a result of time lost from work.

*I have gotten a support which has been through talks and also monetary support from my friends (Private hospital participant 3 with breast cancer).*

*Finances required has been too much then the other burden is that I am not working ….. (Public hospital participant number 5 with breast cancer).*

*When I went to start the drugs, it wasn*’*t it cheap! for I didn*’*t have the money. I went there and I was prescribed the drugs but I didn*’*t have the money. (Public hospital Participant number with 4 breast cancer).*

*It is not really what the disease per se but the expense, it is a very expensive disease to treat (Private hospital Participant number with 7 breast cancer)*

The cost of cancer was variable, but women felt that being cared for by others; either through provision of material things or necessary information helped them cope in a difficult situation. Even when financial support was unavailable, the mere presence of a friend and positive encouragement was seen as helpful*.*

*Yes, even if you are not given me something there is a something that you are giving. The encouragement (Private hospital participant 6 with breast cancer).*

#### Social support

Kenyan woman receive support in a variety of ways and from many different sources. Support in this study is adopted from the definition by Katapodi *et al* [20], who describe it as the interchange of services between at least two people, the provider and the recipient, with the intent of enhancing the recipient’s well-being. The need for support was consistently expressed by participants from the point of diagnosis and throughout their treatment journey. Support came from many directions including family, friends, the clinical team and other patients. Husbands were referred to most frequently and identified as a major source of family support. Family support included that received from spouse, sisters, children, friends and other relatives.

*I have received a great support from my husband, and the way he has viewed it is that now that I have breast cancer, he is positive, and he is always encouraging me (Private hospital participant 3 with breast cancer).*

*So, I had my husband talk about to me because he was the only person that I was talking to (Private hospital participant 10 with cervical cancer).*

*My family members I can say it*’*s my husband because actually we took the task the two of us we used to comfort one another (Private hospital participant 1 with breast cancer).*

Sisters, children and in-laws were also highlighted as key supports within participant’s family networks.

*They both comforted me. And this last-born son who is a police, who is working with the central bank he told me, most of his own colleagues are positive (Private hospital participant 2 with breast cancer).*

*They are my sisters. Do you know they are sisters, they can understand? (Private hospital participant 10 with cervical cancer).*

*And that why when I was done counseling, my mother in-law called me and told me that so and so from our village they do not have their breast for thirty years, another has forty years and at that time I told God instead of having all this stress….my pressure was high, and for all those that I have be told that they have been removed their breasts they are alive and you cannot know it. (Public hospital participant 2 with breast cancer).*

Friends also provided much support with indications that folks rallied around after a diagnosis became known and the need for support was understood.

*Ok. What has happened after the diagnosis I have gotten people becoming closer and closer to me, and we have…….they have been encouraging me and sharing a lot to me. Most of the people I can say that they are people who have shared with me, or those who know they have come closer to me. They have not left me alone, and distanced themselves from me but they have come to share. (Public hospital participant 1 with breast cancer).*

*My friends who we go to church with, and because where I come from we have some women who have under done through this cancer treatment they have lost their breast so when we are together and we were sharing (Private hospital participant 5 with breast cancer).*

Several participants also highlighted cancer survivor support groups as a helpful source of support.

*And doctor told me lest just go to the support group and when we went there we found about sixty women (Private hospital participant 10 with cervical cancer).*

*Yes it was beneficial. Because if it were not the ones who counseled me by telling me that I have a life to live after the surgery, and I am worthy person here on earth and if you have not finished his work you cannot die. Then I thought my life is worthy (Private hospital participant 1 with breast cancer).*

Participants noted that the way in which people spoke with them was helpful and supportive. Equally, positive encouragement was a valued support.

*The way she talked to me was able to put me at ease (Private hospital participant 7 with breast cancer).*

*Yeah, my friends are positive and thus we encourage each other. We encourage each other on who to be very positive about the disease (Private hospital participant 9 with cervical cancer).*

Even when financial support is unavailable, the presence of a friend and positive encouragement is seen as helpful.

*Yes, even if you are not given me something there is a something that you are giving. The encouragement (Private hospital participant 6 with breast cancer).*

In addition to support from others, a number of learned skills, such as meditation, were noted as a key source of support. Importantly, faith was identified as a major support, while finances led to another/separate theme. Both of these themes are identified earlier in this manuscript.

#### Side effects of treatment

The theme of side effects is evident throughout the interviews. In this study, side effects are defined as the symptoms experienced by an individual, including effects on organ systems, sexuality and fatigue [21, 22].

*…the doctor … she told me about the side effects. I didn’t undergo some of the side effects … but … I went and both [bought] wig so that I may be covering my head (Private hospital participant 8 with breast cancer).*

Side effects mentioned most frequently by women include alopecia (hair loss), skin and nail changes, nausea and vomiting and fatigue. The women mentioned alopecia in this way:

*[I] don’t have what my hair. Mmm but what I thank God because [I] am not in pain. What I keep on telling myself [I] am safe only hair (Private hospital participant 7 with breast cancer).*

They became concerned about infection, skin changes and nausea and vomiting:

*I should not be near a sick person. I should not be near many people I always have fresh air while in a room like this, the door should be open. Even I have kept on checking (Private hospital participant 2 with breast cancer).*

*am disturbed when I look at my skin it has started turning black (Private hospital participant 5 with breast cancer).*

*I had actually darkened but now they are coming back to normal. I am thanking God for that (Private hospital participant 7 with breast cancer).*

*You get depressed, you don’t eat, you vomit. The nausea and vomiting … just too weak you can’t [you] eat are just looking at people (Private hospital participant 9 with cervical cancer).*

*When I find myself vomiting I need to take a lot of water … also for food I should not fail to eat … but need to take food in small bits (Private hospital participant 4 with breast cancer).*

Another symptom that was discussed by our study participants was cancer-related fatigue. Fatigue is a ‘distressing, persistent subjective sense of physical, emotional, and/or cognitive tiredness or exhaustion related to cancer or cancer treatment that is not proportional to recent activity and interferes with usual functioning’ [23].

*…there is no treatment for … being fatigued (Private hospital participant 7 with breast cancer).*

*I do just go through what fatigue, but even at my worse am still able to walk. Yes, I feel it, but even at worse I can still [what] go to the school and get back to the house (Private hospital participant 7 with breast cancer).*

Women spoke about the importance of education about side effects and the impact of that education on them. They said the following:

*Because when I started the first chemo they took me all through the information very well the side effects and everything so I had the information (Private hospital participant 3 with breast cancer).*

*Because they told me about everything, before I could take my first, my first chemo (Private hospital participant 8 with breast cancer).*

*We need to be taught. Like the TV screen out there sometimes it shows some programmes … Instead it should be showing us programs related to cancer… (Private hospital participant 2 with breast cancer).*

*… I think I was given what a booklet … what talked about the side effects (Private hospital participant 7 with breast cancer).*

## Discussion

Majority of the women received the cancer diagnosis with shock and disbelief. These feelings were, however, countered by a hope that the cancer was a temptation that would be overcome if they held strongly to their faith in God. Consistent with prior research, whereas access to health information is an important factor for people to make decisions [24], faith and religion also play a major role in how participants make decisions.

The expression of faith amongst participants is out of the actual scope of the study that was exploring sources of cancer information; yet it has an impact on participants’ experience and perceptions of the information. Most notably, these findings highlight how faith played a role indirectly by giving them inner peace.

People who have a strong faith tend to live healthier lives since they experience higher levels of wellbeing, life satisfaction and happiness [25]. Additionally, they have better social support established through the religious group fellowship meetings, usually twice a week (Sundays and midweek). Among many East Africans, religion is communal, thus the frequent meetings of groups ascribing to the same faith. Faith also deters risky behaviours, thus people with a strong faith are highly not to smoke, drink or engage in risky sexual practices. Such people also have better compliance to medication since they believe that God heals them through the medicine. These findings reflect those of Canada *et al* [26], who reported that faith had an indirect effect on physical and mental functioning. Among the Christians, a common belief is that as an individual you have to play your part, and then God completes it. This part is taking medication and adhering to instructions from the healthcare providers; God then completes the work of healing.

The literature reports a strong relationship between faith and quality of life. There is evidence from studies that cancer survivors who profess a faith report better quality of life [26]. However, the specific contribution of faith is not clearly understood. From this study, women expressed the comfort they got by believing in God, which reduced the burden of the cancer diagnosis and suffering caused by the side effects of chemotherapy and radiotherapy. This is similar to the findings of Polonyi *et al* [13], who through a qualitative study explored African American breast cancer survivors’ quality-of-life in the post-treatment period with a focus on social and spiritual well-being. In that study, a shared characteristic among the participants was the confidence the relationship with God gives to everyday living encounters. Their expressions exhibited confidence in God and confirmation that God was with them as they narrated their cancer experiences.

These results need further scrutiny, but there are some immediately dependable conclusions that faith and spiritual wellbeing have a positive impact on the patient’s cancer journey. Further comfort is perceived from the women’s description of how God would guide, protect and eventually heal them of the cancer. Some were confident that even if they died, they were close to God and would go to heaven. This study supports evidence from previous studies that people who believed in a higher being coped better with cancer [27]. There are several possible explanations for this: God guides them through the management of cancer, God provides for their treatment and He is in control of their lives and God had the healing powers. Surprisingly, even the uncertainty caused by a cancer diagnosis was accepted as God’s way. A likely explanation is that most religious groups believe that adhering to their faith and religion is not easy; thus they have to withstand trials and tribulations, which come in the form of illnesses.

It is noteworthy that in addition to faith, women in the study also drew strength from their support network consisting of their spouse, immediate family and women friends or ‘sisters’. A possible explanation is that these networks may have given women the added strength to cope with the societal stigma of having cancer and with the obvious side effects such as alopecia. Additionally, the women received information from their social networks which helped them cope. Although this finding cannot be extrapolated to all participants, for example, the public hospital participant 5 with breast cancer had poor social networks and encountered challenges during treatment. This is because social networks play other roles, such as providing a contribution towards the finances needed for cancer treatment. These findings agree with those of previous studies [28–30] which laud the contribution of social networks to cancer patients’ wellbeing.

When the participants learnt of their cancer status, they despaired. This concurred with the findings of Kyota & Kanda [31], on experiences of terminally ill cancer patients. Participants used religious faith [32] in coping with the diagnosis and depended on it for comfort. The difficulty and lengthy treatment process for the cancer disease plus the treatment cost increase the feeling of fear and despair. Cancer is an expensive disease to treat. Large financial demands are placed on individuals and families in meeting the treatment cost and diagnosis for cancer.

Moreover, participants in this study, having had cancers of the cervical and breast, which are termed as ‘private parts’, presented with fear for they were not aware of how their partners and society would perceive their cases. The possibility of losing or have a medical procedure done to the organs with unprecedented outcomes added stigma on patients. Fear of death cut across the transcripts in this study. Fear seemed to be an original theme for most women, but the fear of death was aggravated after receipt of diagnostic results [33] state that thoughts of death and anxiety are common phenomena expressed by patients with terminal illness.

The social system supporting cancer patients is of great significance in ensuring the patient access health care and pulls through the treatment process. Close family members like husbands, children, in-laws and siblings were well noted to have formed the patient support system. They offered emotional and financial support to cancer patients. This helps to improve the patient’s emotional well-being [33].

These findings revealed that cancer care and management are a costly endeavour that requires extensive testing prior to diagnosis confirmation and throughout the treatment journey. Even after the rigorous diagnostic procedures, cancer patients were apprehensive of the results of their diagnosis. These results were devastating for the participants, creating anxiety and a sense of fatalism. Most of the participants verbalised that being diagnosed with breast cancer was a death sentence. Better mental preparation for women diagnosed with breast or cervical cancer is required prior to disclosing the cancer diagnosis. The findings support findings from previous studies [34–36] which examined the factors that affect cancer awareness, diagnosis and treatment. According to the results of this study, women with breast or cervical cancer have a number of needs, with financial needs being the most important, accompanied by a need for psychosocial and spiritual support. Consequently, approaches focusing on the core information needs, such as financial, psychosocial, spiritual, emotional and informational needs at the time of diagnosis and during care, should be established.

The side effects documented in the literature include effects on organ systems, sexuality and fatigue [21, 22]. The women also talked about the importance of education about side effects, stating that information helped them know what to expect and afforded them a level of control in an otherwise uncertain cancer treatment journey. Alopecia, partial or complete hair loss from areas of the body which normally have hair growth, is cited as ‘one of the most common and distressing effects of chemotherapy’ [37]. This type of hair loss can cause such fear that patients actually decline treatment. Women in this study expressed sadness at losing their hair and the effect it had on their significant others. A participant talked about the effect of alopecia on her young son, and this was distressing. Bone marrow suppression, also known as myelosuppression, encompasses neutropenia, anaemia and thrombocytopenia, all of which can result in devastating physical symptoms necessitating hospitalisation and treatment delay [21]. Women expressed fear of getting an infection, ‘I should not be near a sick person. I should not be near many people I always have fresh air while in a room like this; the door should be open’. The participants feared getting secondary infections, but they drew strength in the word of God to cope. These findings seem to suggest that other factors are crucial in the treatment journey and not just information. Despite all the challenges of managing side effects, study participants who were proactively educated about side effects were somewhat better prepared, coped better and overall had a better experience.

The importance of proactive patient education is also reinforced by study findings [21, 22, 38]; women reported the impact of this education. Future studies can explore the most effective means of providing this education and partner with cancer survivors to develop patient-centred, contextually relevant educational materials.

### Study limitations

The small purposive sample poses potential limitations, and the findings from this study are not generalisable to Kenyan women who are diagnosed with breast and cervical cancer. There might be information bias and/or recall bias regarding disease stage and presenting symptoms. This study was conducted in Nairobi, a large urban centre, and may not necessarily capture the experiences of women living in other smaller centres or villages. These women may not necessarily have access to screening programmes and the level of tertiary care available in Nairobi.

## Conclusion

This study suggests that individual HL is not the ultimate in decision-making and involvement. Still, other issues such as fear, faith, social support and finances contribute to the ability and capacity to use information. The study findings will inform educational programme content for nurses involved in primary health care settings, specialist oncology settings and specifically patients diagnosed with breast or cervical cancer.

## List of abbreviations

HL, Health literacy; NACOSTI, National Commission for Science, Technology, and Innovation; NHIF, National Hospital Insurance Fund.

## Conflicts of interest

None of the authors have conflicts of interest to declare.

## Funding

A grant funded study by CRDFGLOBAL Beginning Investigator Grant for Catalytic Research (BIG CAT) Initiative.

## Figures and Tables

**Table 1.** Participant characteristics.

Characteristic (*N* = 18)	Description	100.00% (*N* = 18)
Age	Average age	45.33 years
Religion	Muslim	(5.56%) *N* = 1
	Christian	(94.44%) *N* = 17
Education	Primary level education and below	(11.11%) *N* = 2
	Secondary school level drop out	(5.56) *N* = 1
	Secondary school level	(50.00%) *N* = 9
	College/university level	(33.33%) *N* = 6
Marital status	Single	(5.56%) *N* = 1
	Married	(83.33%) *N* = 15
	Separated	(11.11%) *N* = 2
Type of marriage	Monogamous	(77.78%) *N* = 14
	Polygamous	(5.56%) *N* = 1
	No response	(16.67%) *N* = 3
Work status	Full time	(50.00%) *N* = 9
	Part time	(11.11%) *N* = 2
	Retired	(5.56%) *N* = 1
	Not working	(33.33%) *N* = 6
Wage level	High	(11.11%) *N* = 2
	Middle	(50.00%) *N* = 9
	Low	(38.89%) *N* = 7
Residence	Rural	(50.00%) *N* = 9
	Urban	(50.00%) *N* = 9

**Table 2.** Distribution of patients across the public and private hospitals.

	Private (Aga Khan)	Public (Kenyatta)
Breast cancer	6	5
Cervical cancer	4	3
Total	10	8

## Appendix A. Semi-structured interview guide.

Interview questions

1. Events leading to diagnosis
  1. Tell me about how long you have been unwell
  2. When did you first go to the clinic or the hospital and why ______ hospital.
  3. What made you want to see the doctor?
2. Current understanding of illness, treatment & medicines
  1. What did the doctor/nurse explain to you when you went to the hospital?
  2. Tell me what you understand/understood about what they said about what you were experiencing and what they said might be wrong?
  3. Can you tell me in your own words what you think is wrong?
  4. Did they give you any instruction or medicines to take home? Can you show them to me and tell me what you understand about the medicines/instructions?
3. Cultural beliefs
  1. Apart from what the doctor said will help you, do you have any other beliefs about things that will help you get better?
  2. Are you also consulting a traditional healer and how have they explained things?
4. Reflections
  1. What could the doctor/nurse have done differently to help you better understand your illness/medicines/instructions?

Post interview summary

ID: _____________________________________

Date: ___________________________________

Interviewer: ______________________________

Length of interview: ________________________

Location: _________________________________

Type of interview (circle one): Initial first follow-up second follow-up

Detailed questions (Interviewer to fill out notes):

How did the woman appear to present (comfortable, uncomfortable)?

Who was present at the interview? (specific family members, care-givers, the woman was alone)

Was the woman ready to engage in a conversation? (Nervous at first, reluctant to engage, eager to engage)

Items that came up during the interview that should be followed up at the next interview.

## Appendix B. PIS and informed consent form.

**Informed consent form for HL study, Nairobi, Kenya**

This informed consent form is for women with cancers of the breast or cervical who we are inviting to participate in research titled, ‘Cancer related health literacy status, information and educational needs of patients diagnosed with cancers of the cervical and breast in Kenya’.

**Name of principle investigator: **Dinah Kassaman

**Name of organisation: **Aga Khan University School of Nursing and Midwifery

**Contact information: **diana.kassaman@aku.edu Telephone: 0722408019

sharon.brownie@aku.edu Telephone: 0733247174

Tayreez.mushani@aku.edu Telephone: 0733400474

**This informed consent form has two parts:**

- **Information sheet (to share information about the study with you)**
- **Consent form (for signatures if you choose to participate)**

**You will be given a copy of the full informed consent form**

**Study title**

Cancer related health literacy: a qualitative research study of the information and educational needs of patients diagnosed with cancers of the breast or cervical in Kenya.

**Patient information sheet (PIS)**

**Study title:** Cancer related health literacy status, information and educational needs of patients diagnosed with cancers of the cervical and breast in Kenya.

**Introduction**

My name is Dinah Kassaman and I am a lecturer at the Aga Khan University School of Nursing and Midwifery in Nairobi. I would like to invite you to take part in a research study. Before you decide, I need you to understand why the research is being done and how you will be involved. Before you decide, you can talk to anyone you feel comfortable with about the research. If there are any words on this form that you do not understand, please ask us and we will be happy to help. We are doing research to identify the type of information that is given to women with cancer and how the women understand this information. If you have questions later, you can ask me or anyone on my team. My contact information is provided above.

**Purpose of the research**

The number of women with breast/cervical cancer is increasing in Kenya and the government is concerned about it. We want to understand how women with cancer understand the information that is given to them about their illness and treatment. This knowledge will help us to improve the information provided from health care professionals.

**Participant selection**

You are being invited to take part in this research because you have been diagnosed with cancer of the cervical/breast and you are on treatment. Your experiences and how information was given to you are what this study seeks to hear. We feel that by sharing your experience, you can help us to better understand the type of information that is helpful for patients with cancer.

**Voluntary participation**

Taking part in this research is completely voluntary. We will describe the study and go through the information sheet which we will give you. We will then ask you to sign a consent form to show that you have agreed to take part. If you decided to take part in the study, you are free to withdraw at any time without giving a reason; this will not affect your treatment in any way and all data up to the point that you withdraw will be stored for a minimum of 5 years from the hand in date.

**What will happen to you if you take part?**

I will explain to you about the study in the cancer clinic, then I will give you an information sheet which is detailed and a consent form. You will go home with the documents, and after reading and agreeing to participate in the study, you will sign the consent form and return it to the clinic on your next visit. If you agree to participate, the research assistant will call you so that the time and venue of the interview can be planned and agreed upon. The research will take place over 18 months in total. During that time, we will visit you three times for interviewing. If you accept, you will be invited to participate in three (3) separate interviews which will last approximately 30–60 minutes. The first interview will be decided by you, followed by two more interviews of 6 weeks’ interval. During the interview, I or another interviewer will sit down with you in a comfortable place either at the hospital or if it is better for you, we can also do the interview at your home. We will ask you a series of questions which we have prepared. If you do not wish to answer a particular question during the interview, you can tell the interviewer and they will move to the next question. No one else but the interviewer will be present unless you would like someone else to be there. We will tape record the information so that we can listen to it later and transfer it to text format. No one else except the research team will be able to see this information. No one will be identified by name on the tape. The recording will be kept in a locked cupboard and will be destroyed after the research is finished.

**Risks**

There are no risks to you if you participate in this research but, if we ask you a question that makes you feel uncomfortable, you can choose not to answer the question. In case you get distressed as you recall the events surrounding the cancer diagnosis and treatment, there will be counselling services available and we will appropriately refer you for psychological support if you agree.

Details of psychological support service can be found at the end of this information sheet.

**Possible benefits**

We cannot promise that the study will be of direct benefit to you, but the information from this research will help to increase the understanding of the type of health information to be given to women with cancer.

**Will my taking part in the study be kept confidential?**

All information which is collected from you during the course of the research will be anonymised, and any information about you which leaves the university will have your name and address removed so that you cannot be recognised. If, however, you would prefer to be credited for your contribution, I will be happy to ensure that you are clearly named and referenced.

Further to this:

- Your data will be stored safely, specifically:
  - Individual participant research data, such as questionnaires/interview scripts will be anonymous and given a research code, known only to the researcher.
  - A master list identifying participants to the research codes data will be held on a password protected computer accessed only by the researcher.
  - Hard paper/taped data will be stored in a locked cabinet, within locked office, accessed only by researcher.
  - Electronic data will be stored on a password protected computer known only by researcher.
- Your data will be used solely for the purposes of this study.
- Your data will be accessible only by authorised persons such as researchers within the team, supervisors and regulatory authorities (Kenya Ethics committees and the Kenya National Council of Science and Technology (NACOSTI).
- Your data will be retained for a maximum of 3 years (after the hand in date) before being disposed of in a controlled manner.

Also note that any information about you which leaves the university will have your personal details removed so that you cannot be recognised. Results would be published in research journals or presented in conferences or elsewhere without disclosing participants’ names.

‘I am aware that if I reveal anything that is harmful to self or others, the researcher will have to share that information with the appropriate authorities’.

**What if there is a problem?**

In case you have a concern about any aspect of the study, you should ask to speak to the researcher (Dinah Kassaman 0722408019 or Tayreez Mushani 0733400474) who will do their best to answer your questions. If your issue is not sorted out and you wish to complain formally, you can do this by contacting the Research Supervisor (Professor Allison Brettle, Telephone number: 0161 295 0447) or the chairman of Research committee, Aga Khan University, Nairobi (Professor William Macharia 020 3 740000 EXT 2148/1136).

**What will happen if I do not carry on with the study?**

If you withdraw from the study, all tape recorded interviews collected from you, to that date will be destroyed and your name removed from the study files; we may however need to use the data collected up until your withdrawal.

**What will happen to the results of the research study?**

The study findings will be reported through publications in journals – one in the field of cancer nursing and another in an education journal. In addition, the Aga Khan University has links with national news reporters and newspapers and an article will be drafted for press release. Study investigators will also share findings by making formal presentations at Cancer nursing conferences. Results will also be shared with local health care professionals through the weekly seminar series on ongoing faculty research activities. You will not be identified in any report or publication.

**Who is organising or sponsoring the research?**

This research has been funded by CRDFGLOBAL under BIG CAT initiative.

For Psychological support, call:

Mrs Mary Gitau

Telephone: +254722753473/731888066

For further information or to ask questions contact:

Professor Alison Brettle

Professor in Health Information and Evidence Based Practice|

School of Health and Society, University of Salford,

Mary Seacole Building, Frederick Road, Salford, M6 6PU

Tel: 0161 295 0447, Email: a.brettle@salford.ac.uk

**Table:**

Consent form for HL study Please tick the appropriate boxes		
**Taking part**	**Yes**	**No**
I have read and understood the project information sheet dated 01/12/2017.	□	□
I have been given the opportunity to ask questions about the project.	□	□
I agree to take part in the project. Taking part in the project will include being interviewed and audio recorded.	□	□
I understand that my taking part is voluntary; I can withdraw from the study at any time and I do not have to give any reasons for why I no longer want to take part.	□	□
**Use of the information I provide for this project only**		
I understand my personal details such as phone number and address will not be revealed to people outside the project.	□	□
I understand that my words may be quoted in publications, reports, web pages and other research outputs.	□	□
*Please choose* ***one**** of the following two options:* I would like my real name used in the above. I would **not** like my real name to be used in the above.	□ □	
**Use of the information I provide beyond this project**		
I agree for the data I provide to be archived at the UK Data Archive.	□	□
I understand that other authenticated researchers will have access to this data only if they agree to preserve the confidentiality of the information as requested in this form.	□	□
I understand that other authenticated researchers may use my words in publications, reports, web pages and other research outputs, only if they agree to preserve the confidentiality of the information as requested in this form.	□	□
**So we can use the information you provide legally**		
I agree to assign the copyright I hold in any materials related to this project to (Dinah Makuba Kassaman)	□	□

__________ __________ __________

Name of Participant Printed Signature Date

__________ __________ __________

Name of Researcher Printed Signature Date
